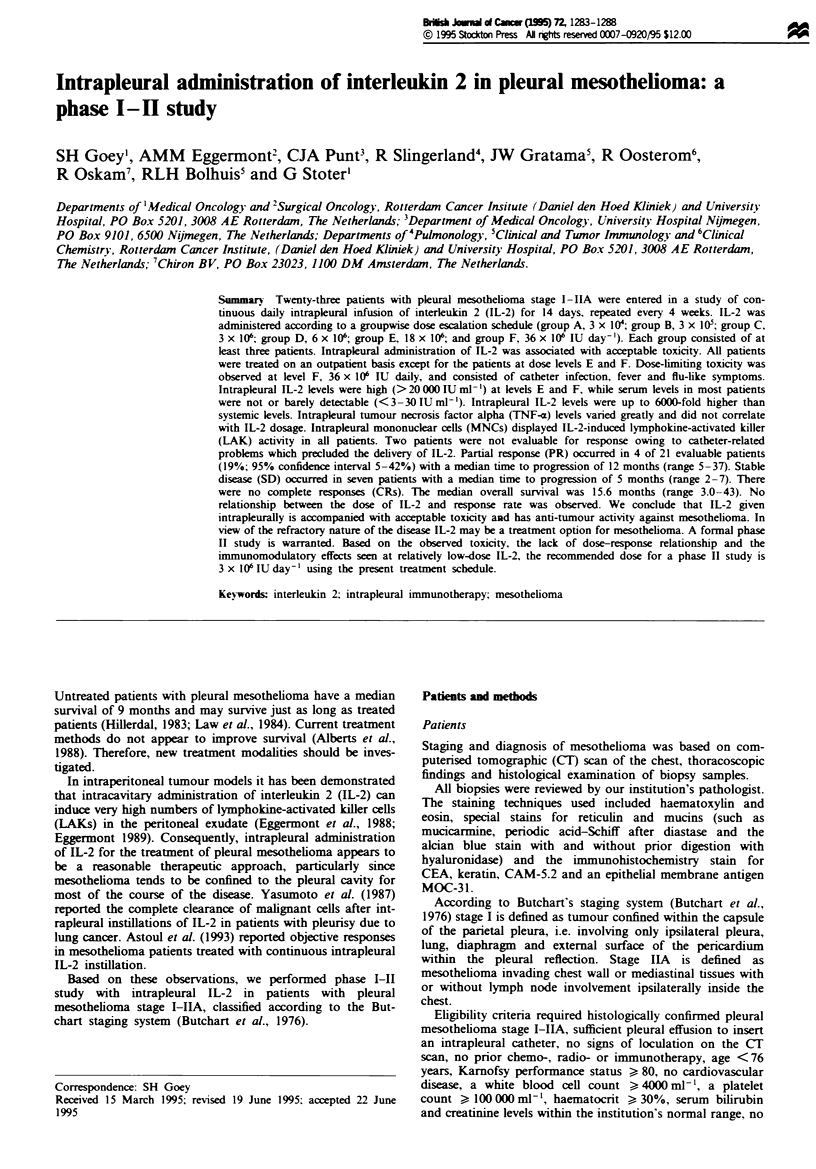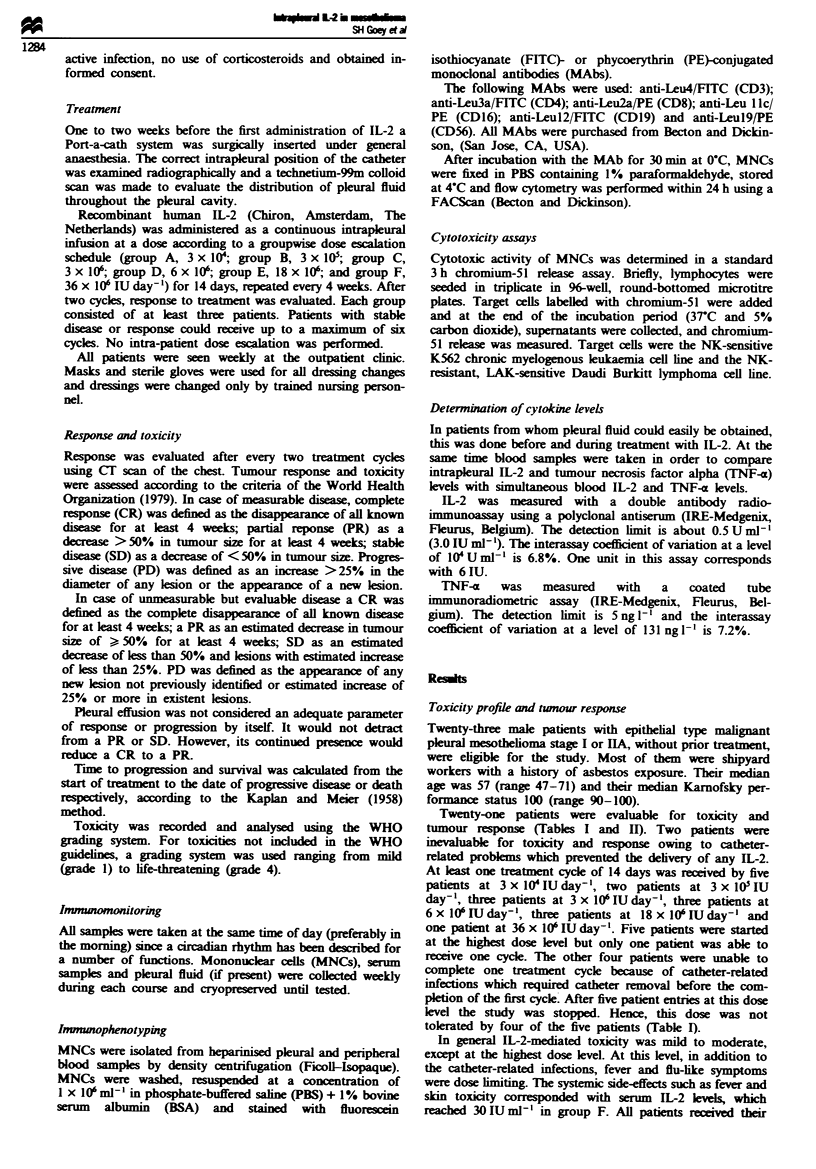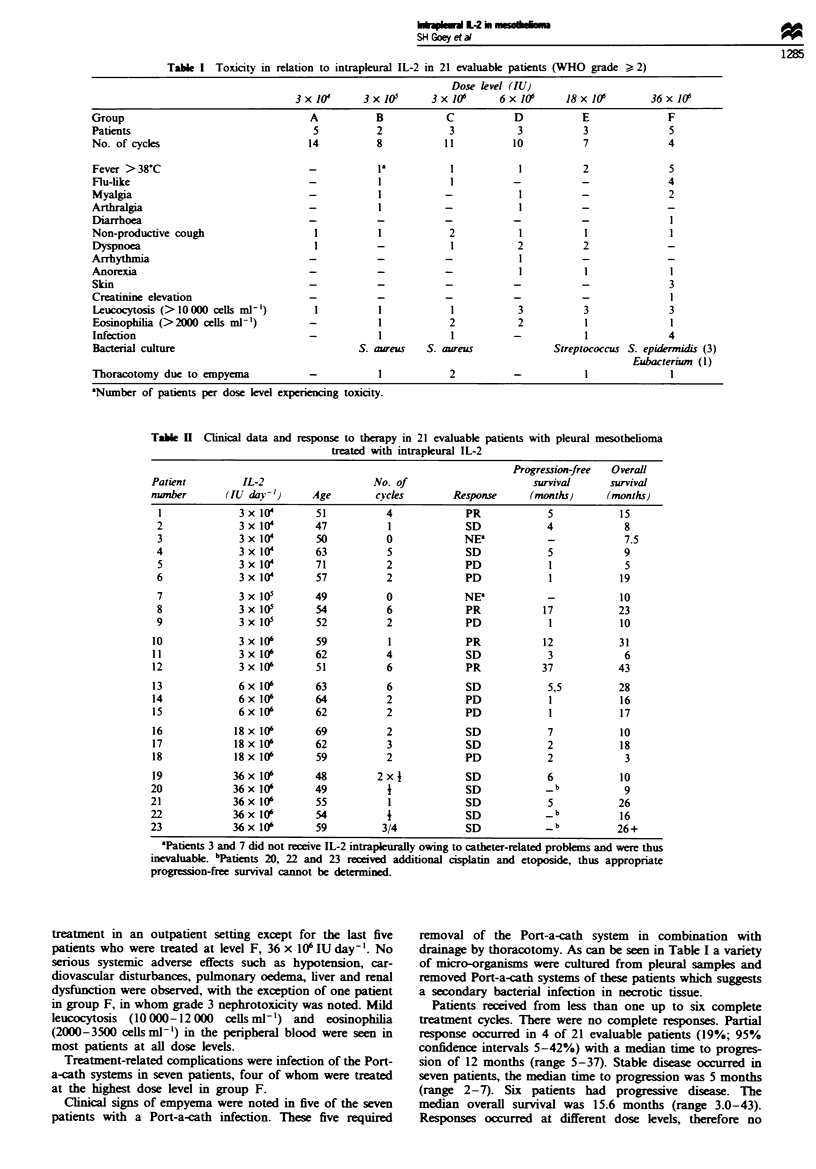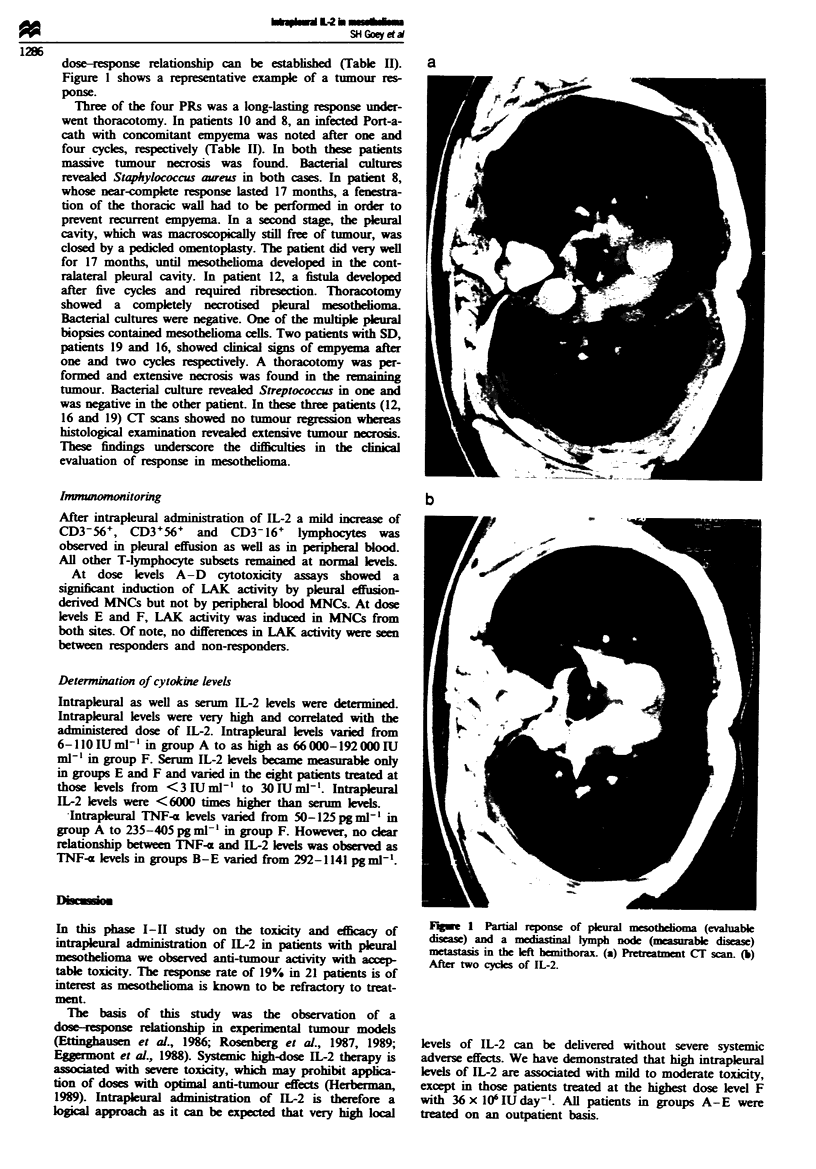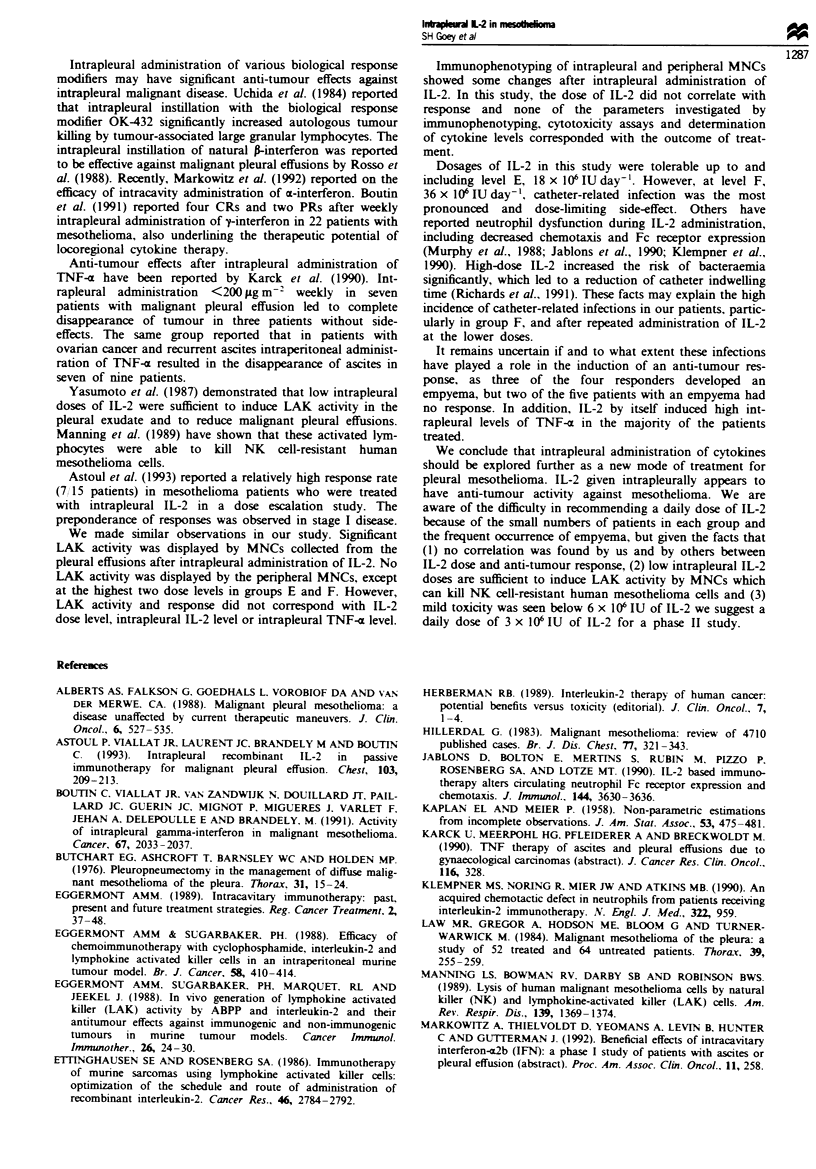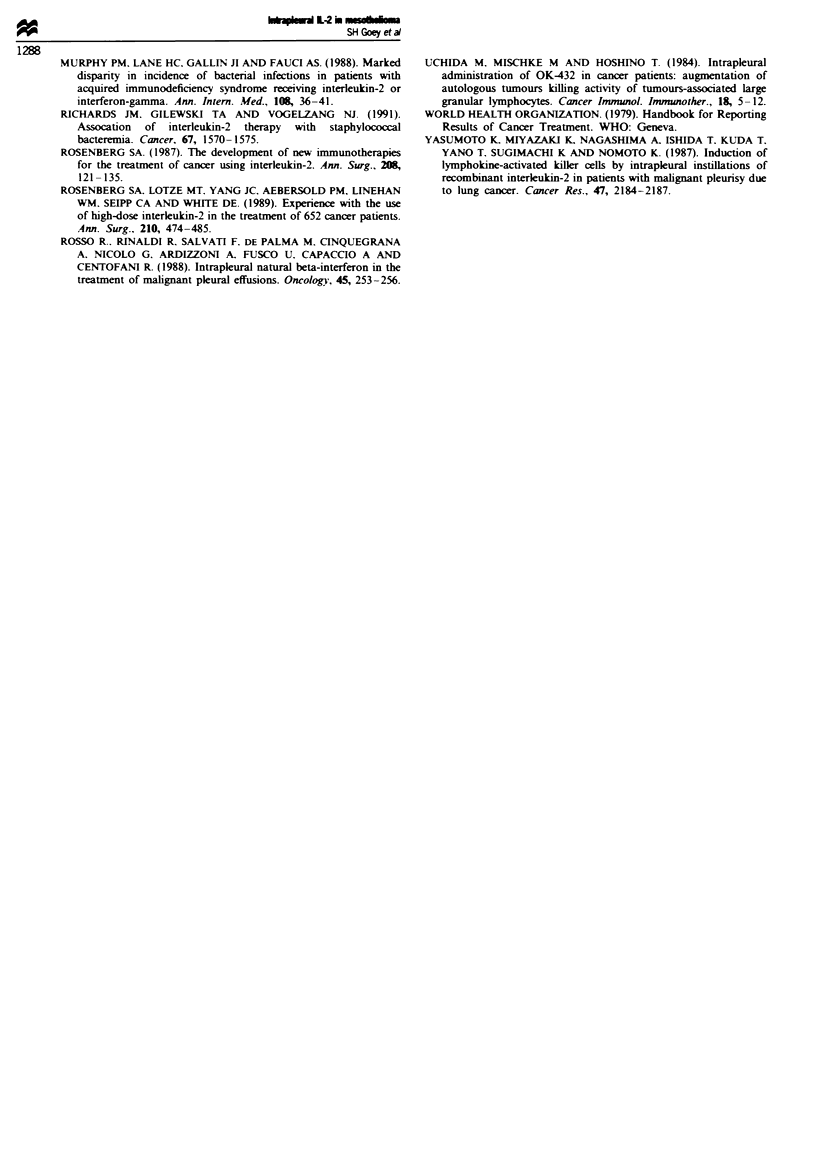# Intrapleural administration of interleukin 2 in pleural mesothelioma: a phase I-II study.

**DOI:** 10.1038/bjc.1995.501

**Published:** 1995-11

**Authors:** S. H. Goey, A. M. Eggermont, C. J. Punt, R. Slingerland, J. W. Gratama, R. Oosterom, R. Oskam, R. L. Bolhuis, G. Stoter

**Affiliations:** Department of Medical Oncology, Rotterdam Cancer Institute (Daniel den Hoed Kliniek), The Netherlands.

## Abstract

**Images:**


British Journal of Cancer (1995) 72, 1283-1288

© 1995 Stockton Press All rights reserved 0007-0920/95 $12.00

### Intrapleural administration of interleukin 2 in pleural mesothelioma: a phase I-II study

SH Goey[1], AMM Eggermont[2], CJA Punt[3], R Slingerland[4], JW Gratama[5], R Oosterom[6], R Oskam[7], RLH Bolhuis[5] and G Stoter[1]

*Departments of [1]Medical Oncology and [2]Surgical Oncology, Rotterdam Cancer Insitute (Daniel den Hoed Kliniek) and University Hospital, PO Box 5201, 3008 AE Rotterdam, The Netherlands; [3]Department of Medical Oncology, University Hospital Nijmegen, PO Box 9101, 6500 Nijmegen, The Netherlands; Departments of [4]Pulmonology, [5]Clinical and Tumor Immunology and [6]Clinical Chemistry, Rotterdam Cancer Institute, (Daniel den Hoed Kliniek) and University Hospital, PO Box 5201, 3008 AE Rotterdam, The Netherlands; [7]Chiron BV, PO Box 23023, 1100 DM Amsterdam, The Netherlands.*

**Summary** Twenty-three patients with pleural mesothelioma stage I-IIA were entered in a study of continuous daily intrapleural infusion of interleukin 2 (IL-2) for 14 days, repeated every 4 weeks. IL-2 was administered according to a groupwise dose escalation schedule (group A, $3 \times 10^4$; group B, $3 \times 10^5$; group C, $3 \times 10^6$; group D, $6 \times 10^6$; group E, $18 \times 10^6$; and group F, $36 \times 10^6$ IU day$^{-1}$). Each group consisted of at least three patients. Intrapleural administration of IL-2 was associated with acceptable toxicity. All patients were treated on an outpatient basis except for the patients at dose levels E and F. Dose-limiting toxicity was observed at level F, $36 \times 10^6$ IU daily, and consisted of catheter infection, fever and flu-like symptoms. Intrapleural IL-2 levels were high (>20 000 IU ml$^{-1}$) at levels E and F, while serum levels in most patients were not or barely detectable (<3-30 IU ml$^{-1}$). Intrapleural IL-2 levels were up to 6000-fold higher than systemic levels. Intrapleural tumour necrosis factor alpha (TNF-α) levels varied greatly and did not correlate with IL-2 dosage. Intrapleural mononuclear cells (MNCs) displayed IL-2-induced lymphokine-activated killer (LAK) activity in all patients. Two patients were not evaluable for response owing to catheter-related problems which precluded the delivery of IL-2. Partial response (PR) occurred in 4 of 21 evaluable patients (19%; 95% confidence interval 5-42%) with a median time to progression of 12 months (range 5-37). Stable disease (SD) occurred in seven patients with a median time to progression of 5 months (range 2-7). There were no complete responses (CRs). The median overall survival was 15.6 months (range 3.0-43). No relationship between the dose of IL-2 and response rate was observed. We conclude that IL-2 given intrapleurally is accompanied with acceptable toxicity and has anti-tumour activity against mesothelioma. In view of the refractory nature of the disease IL-2 may be a treatment option for mesothelioma. A formal phase II study is warranted. Based on the observed toxicity, the lack of dose-response relationship and the immunomodulatory effects seen at relatively low-dose IL-2, the recommended dose for a phase II study is $3 \times 10^6$ IU day$^{-1}$ using the present treatment schedule.

**Keywords:** interleukin 2; intrapleural immunotherapy; mesothelioma

Untreated patients with pleural mesothelioma have a median survival of 9 months and may survive just as long as treated patients (Hillerdal, 1983; Law *et al.*, 1984). Current treatment methods do not appear to improve survival (Alberts *et al.*, 1988). Therefore, new treatment modalities should be investigated.

In intraperitoneal tumour models it has been demonstrated that intracavitary administration of interleukin 2 (IL-2) can induce very high numbers of lymphokine-activated killer cells (LAKs) in the peritoneal exudate (Eggermont *et al.*, 1988; Eggermont 1989). Consequently, intrapleural administration of IL-2 for the treatment of pleural mesothelioma appears to be a reasonable therapeutic approach, particularly since mesothelioma tends to be confined to the pleural cavity for most of the course of the disease. Yasumoto *et al.* (1987) reported the complete clearance of malignant cells after intrapleural instillations of IL-2 in patients with pleurisy due to lung cancer. Astoul *et al.* (1993) reported objective responses in mesothelioma patients treated with continuous intrapleural IL-2 instillation.

Based on these observations, we performed phase I-II study with intrapleural IL-2 in patients with pleural mesothelioma stage I-IIA, classified according to the Butchart staging system (Butchart *et al.*, 1976).

Correspondence: SH Goey
Received 15 March 1995; revised 19 June 1995; accepted 22 June 1995

### Patients and methods

### *Patients*

Staging and diagnosis of mesothelioma was based on computerised tomographic (CT) scan of the chest, thoracoscopic findings and histological examination of biopsy samples.

All biopsies were reviewed by our institution's pathologist. The staining techniques used included haematoxylin and eosin, special stains for reticulin and mucins (such as mucicarmine, periodic acid-Schiff after diastase and the alcian blue stain with and without prior digestion with hyaluronidase) and the immunohistochemistry stain for CEA, keratin, CAM-5.2 and an epithelial membrane antigen MOC-31.

According to Butchart's staging system (Butchart *et al.*, 1976) stage I is defined as tumour confined within the capsule of the parietal pleura, i.e. involving only ipsilateral pleura, lung, diaphragm and external surface of the pericardium within the pleural reflection. Stage IIA is defined as mesothelioma invading chest wall or mediastinal tissues with or without lymph node involvement ipsilaterally inside the chest.

Eligibility criteria required histologically confirmed pleural mesothelioma stage I-IIA, sufficient pleural effusion to insert an intrapleural catheter, no signs of loculation on the CT scan, no prior chemo-, radio- or immunotherapy, age <76 years, Karnofsy performance status ⩾ 80, no cardiovascular disease, a white blood cell count ⩾ 4000 ml$^{-1}$, a platelet count ⩾ 100 000 ml$^{-1}$, haematocrit ⩾ 30%, serum bilirubin and creatinine levels within the institution's normal range, no active infection, no use of corticosteroids and obtained informed consent.

*Treatment*

One to two weeks before the first administration of IL-2 a Port-a-cath system was surgically inserted under general anaesthesia. The correct intrapleural position of the catheter was examined radiographically and a technetium-99m colloid scan was made to evaluate the distribution of pleural fluid throughout the pleural cavity.

Recombinant human IL-2 (Chiron, Amsterdam, The Netherlands) was administered as a continuous intrapleural infusion at a dose according to a groupwise dose escalation schedule (group A, $3 \times 10^4$; group B, $3 \times 10^5$; group C, $3 \times 10^6$; group D, $6 \times 10^6$; group E, $18 \times 10^6$; and group F, $36 \times 10^6$ IU day$^{-1}$) for 14 days, repeated every 4 weeks. After two cycles, response to treatment was evaluated. Each group consisted of at least three patients. Patients with stable disease or response could receive up to a maximum of six cycles. No intra-patient dose escalation was performed.

All patients were seen weekly at the outpatient clinic. Masks and sterile gloves were used for all dressing changes and dressings were changed only by trained nursing personnel.

*Response and toxicity*

Response was evaluated after every two treatment cycles using CT scan of the chest. Tumour response and toxicity were assessed according to the criteria of the World Health Organization (1979). In case of measurable disease, complete response (CR) was defined as the disappearance of all known disease for at least 4 weeks; partial reponse (PR) as a decrease >50% in tumour size for at least 4 weeks; stable disease (SD) as a decrease of <50% in tumour size. Progressive disease (PD) was defined as an increase >25% in the diameter of any lesion or the appearance of a new lesion.

In case of unmeasurable but evaluable disease a CR was defined as the complete disappearance of all known disease for at least 4 weeks; a PR as an estimated decrease in tumour size of ≥50% for at least 4 weeks; SD as an estimated decrease of less than 50% and lesions with estimated increase of less than 25%. PD was defined as the appearance of any new lesion not previously identified or estimated increase of 25% or more in existent lesions.

Pleural effusion was not considered an adequate parameter of response or progression by itself. It would not detract from a PR or SD. However, its continued presence would reduce a CR to a PR.

Time to progression and survival was calculated from the start of treatment to the date of progressive disease or death respectively, according to the Kaplan and Meier (1958) method.

Toxicity was recorded and analysed using the WHO grading system. For toxicities not included in the WHO guidelines, a grading system was used ranging from mild (grade 1) to life-threatening (grade 4).

*Immunomonitoring*

All samples were taken at the same time of day (preferably in the morning) since a circadian rhythm has been described for a number of functions. Mononuclear cells (MNCs), serum samples and pleural fluid (if present) were collected weekly during each course and cryopreserved until tested.

*Immunophenotyping*

MNCs were isolated from heparinised pleural and peripheral blood samples by density centrifugation (Ficoll–Isopaque). MNCs were washed, resuspended at a concentration of $1 \times 10^6$ ml$^{-1}$ in phosphate-buffered saline (PBS) + 1% bovine serum albumin (BSA) and stained with fluorescein isothiocyanate (FITC)- or phycoerythrin (PE)-conjugated monoclonal antibodies (MAbs).

The following MAbs were used: anti-Leu4/FITC (CD3); anti-Leu3a/FITC (CD4); anti-Leu2a/PE (CD8); anti-Leu 11c/PE (CD16); anti-Leu12/FITC (CD19) and anti-Leu19/PE (CD56). All MAbs were purchased from Becton and Dickinson, (San Jose, CA, USA).

After incubation with the MAb for 30 min at 0°C, MNCs were fixed in PBS containing 1% paraformaldehyde, stored at 4°C and flow cytometry was performed within 24 h using a FACScan (Becton and Dickinson).

*Cytotoxicity assays*

Cytotoxic activity of MNCs was determined in a standard 3 h chromium-51 release assay. Briefly, lymphocytes were seeded in triplicate in 96-well, round-bottomed microtitre plates. Target cells labelled with chromium-51 were added and at the end of the incubation period (37°C and 5% carbon dioxide), supernatants were collected, and chromium-51 release was measured. Target cells were the NK-sensitive K562 chronic myelogenous leukaemia cell line and the NK-resistant, LAK-sensitive Daudi Burkitt lymphoma cell line.

*Determination of cytokine levels*

In patients from whom pleural fluid could easily be obtained, this was done before and during treatment with IL-2. At the same time blood samples were taken in order to compare intrapleural IL-2 and tumour necrosis factor alpha (TNF-α) levels with simultaneous blood IL-2 and TNF-α levels.

IL-2 was measured with a double antibody radioimmunoassay using a polyclonal antiserum (IRE-Medgenix, Fleurus, Belgium). The detection limit is about 0.5 U ml$^{-1}$ (3.0 IU ml$^{-1}$). The interassay coefficient of variation at a level of $10^4$ U ml$^{-1}$ is 6.8%. One unit in this assay corresponds with 6 IU.

TNF-α was measured with a coated tube immunoradiometric assay (IRE-Medgenix, Fleurus, Belgium). The detection limit is 5 ng l$^{-1}$ and the interassay coefficient of variation at a level of 131 ng l$^{-1}$ is 7.2%.

### Results

*Toxicity profile and tumour response*

Twenty-three male patients with epithelial type malignant pleural mesothelioma stage I or IIA, without prior treatment, were eligible for the study. Most of them were shipyard workers with a history of asbestos exposure. Their median age was 57 (range 47–71) and their median Karnofsky performance status 100 (range 90–100).

Twenty-one patients were evaluable for toxicity and tumour response (Tables I and II). Two patients were inevaluable for toxicity and response owing to catheter-related problems which prevented the delivery of any IL-2. At least one treatment cycle of 14 days was received by five patients at $3 \times 10^4$ IU day$^{-1}$, two patients at $3 \times 10^5$ IU day$^{-1}$, three patients at $3 \times 10^6$ IU day$^{-1}$, three patients at $6 \times 10^6$ IU day$^{-1}$, three patients at $18 \times 10^6$ IU day$^{-1}$ and one patient at $36 \times 10^6$ IU day$^{-1}$. Five patients were started at the highest dose level but only one patient was able to receive one cycle. The other four patients were unable to complete one treatment cycle because of catheter-related infections which required catheter removal before the completion of the first cycle. After five patient entries at this dose level the study was stopped. Hence, this dose was not tolerated by four of the five patients (Table I).

In general IL-2-mediated toxicity was mild to moderate, except at the highest dose level. At this level, in addition to the catheter-related infections, fever and flu-like symptoms were dose limiting. The systemic side-effects such as fever and skin toxicity corresponded with serum IL-2 levels, which reached 30 IU ml$^{-1}$ in group F. All patients received their

**Table I** Toxicity in relation to intrapleural IL-2 in 21 evaluable patients (WHO grade $\geq 2$)

| | *Dose level (IU)* $3 \times 10^4$ | $3 \times 10^5$ | $3 \times 10^6$ | $6 \times 10^6$ | $18 \times 10^6$ | $36 \times 10^6$ |
|---|---|---|---|---|---|---|
| Group | A | B | C | D | E | F |
| Patients | 5 | 2 | 3 | 3 | 3 | 5 |
| No. of cycles | 14 | 8 | 11 | 10 | 7 | 4 |
| Fever >38°C | – | 1[a] | 1 | 1 | 2 | 5 |
| Flu-like | – | 1 | 1 | – | – | 4 |
| Myalgia | – | 1 | – | 1 | – | 2 |
| Arthralgia | – | 1 | – | 1 | – | – |
| Diarrhoea | – | – | – | – | – | 1 |
| Non-productive cough | 1 | 1 | 2 | 1 | 1 | 1 |
| Dyspnoea | 1 | – | 1 | 2 | 2 | – |
| Arrhythmia | – | – | – | 1 | – | – |
| Anorexia | – | – | – | 1 | 1 | 1 |
| Skin | – | – | – | – | – | 3 |
| Creatinine elevation | – | – | – | – | – | 1 |
| Leucocytosis (>10 000 cells $ml^{-1}$) | 1 | 1 | 1 | 3 | 3 | 3 |
| Eosinophilia (>2000 cells $ml^{-1}$) | – | 1 | 2 | 2 | 1 | 1 |
| Infection | – | 1 | 1 | – | 1 | 4 |
| Bacterial culture | | *S. aureus* | *S. aureus* | | *Streptococcus* | *S. epidermidis* (3) *Eubacterium* (1) |
| Thoracotomy due to empyema | – | 1 | 2 | – | 1 | 1 |

[a]Number of patients per dose level experiencing toxicity.

**Table II** Clinical data and response to therapy in 21 evaluable patients with pleural mesothelioma treated with intrapleural IL-2

| *Patient number* | *IL-2 (IU day$^{-1}$)* | *Age* | *No. of cycles* | *Response* | *Progression-free survival (months)* | *Overall survival (months)* |
|---|---|---|---|---|---|---|
| 1 | $3 \times 10^4$ | 51 | 4 | PR | 5 | 15 |
| 2 | $3 \times 10^4$ | 47 | 1 | SD | 4 | 8 |
| 3 | $3 \times 10^4$ | 50 | 0 | NE[a] | – | 7.5 |
| 4 | $3 \times 10^4$ | 63 | 5 | SD | 5 | 9 |
| 5 | $3 \times 10^4$ | 71 | 2 | PD | 1 | 5 |
| 6 | $3 \times 10^4$ | 57 | 2 | PD | 1 | 19 |
| 7 | $3 \times 10^5$ | 49 | 0 | NE[a] | – | 10 |
| 8 | $3 \times 10^5$ | 54 | 6 | PR | 17 | 23 |
| 9 | $3 \times 10^5$ | 52 | 2 | PD | 1 | 10 |
| 10 | $3 \times 10^6$ | 59 | 1 | PR | 12 | 31 |
| 11 | $3 \times 10^6$ | 62 | 4 | SD | 3 | 6 |
| 12 | $3 \times 10^6$ | 51 | 6 | PR | 37 | 43 |
| 13 | $6 \times 10^6$ | 63 | 6 | SD | 5,5 | 28 |
| 14 | $6 \times 10^6$ | 64 | 2 | PD | 1 | 16 |
| 15 | $6 \times 10^6$ | 62 | 2 | PD | 1 | 17 |
| 16 | $18 \times 10^6$ | 69 | 2 | SD | 7 | 10 |
| 17 | $18 \times 10^6$ | 62 | 3 | SD | 2 | 18 |
| 18 | $18 \times 10^6$ | 59 | 2 | PD | 2 | 3 |
| 19 | $36 \times 10^6$ | 48 | $2 \times \frac{1}{2}$ | SD | 6 | 10 |
| 20 | $36 \times 10^6$ | 49 | $\frac{1}{2}$ | SD | –[b] | 9 |
| 21 | $36 \times 10^6$ | 55 | 1 | SD | 5 | 26 |
| 22 | $36 \times 10^6$ | 54 | $\frac{1}{2}$ | SD | –[b] | 16 |
| 23 | $36 \times 10^6$ | 59 | 3/4 | SD | –[b] | 26+ |

[a]Patients 3 and 7 did not receive IL-2 intrapleurally owing to catheter-related problems and were thus inevaluable. [b]Patients 20, 22 and 23 received additional cisplatin and etoposide, thus appropriate progression-free survival cannot be determined.

treatment in an outpatient setting except for the last five patients who were treated at level F, $36 \times 10^6$ IU $day^{-1}$. No serious systemic adverse effects such as hypotension, cardiovascular disturbances, pulmonary oedema, liver and renal dysfunction were observed, with the exception of one patient in group F, in whom grade 3 nephrotoxicity was noted. Mild leucocytosis (10 000–12 000 cells $ml^{-1}$) and eosinophilia (2000–3500 cells $ml^{-1}$) in the peripheral blood were seen in most patients at all dose levels.

Treatment-related complications were infection of the Port-a-cath systems in seven patients, four of whom were treated at the highest dose level in group F.

Clinical signs of empyema were noted in five of the seven patients with a Port-a-cath infection. These five required removal of the Port-a-cath system in combination with drainage by thoracotomy. As can be seen in Table I a variety of micro-organisms were cultured from pleural samples and removed Port-a-cath systems of these patients which suggests a secondary bacterial infection in necrotic tissue.

Patients received from less than one up to six complete treatment cycles. There were no complete responses. Partial response occurred in 4 of 21 evaluable patients (19%; 95% confidence intervals 5–42%) with a median time to progression of 12 months (range 5–37). Stable disease occurred in seven patients, the median time to progression was 5 months (range 2–7). Six patients had progressive disease. The median overall survival was 15.6 months (range 3.0–43). Responses occurred at different dose levels, therefore no dose–response relationship can be established (Table II). Figure 1 shows a representative example of a tumour response.

Three of the four PRs was a long-lasting response underwent thoracotomy. In patients 10 and 8, an infected Port-a-cath with concomitant empyema was noted after one and four cycles, respectively (Table II). In both these patients massive tumour necrosis was found. Bacterial cultures revealed *Staphylococcus aureus* in both cases. In patient 8, whose near-complete response lasted 17 months, a fenestration of the thoracic wall had to be performed in order to prevent recurrent empyema. In a second stage, the pleural cavity, which was macroscopically still free of tumour, was closed by a pedicled omentoplasty. The patient did very well for 17 months, until mesothelioma developed in the contralateral pleural cavity. In patient 12, a fistula developed after five cycles and required ribresection. Thoracotomy showed a completely necrotised pleural mesothelioma. Bacterial cultures were negative. One of the multiple pleural biopsies contained mesothelioma cells. Two patients with SD, patients 19 and 16, showed clinical signs of empyema after one and two cycles respectively. A thoracotomy was performed and extensive necrosis was found in the remaining tumour. Bacterial culture revealed *Streptococcus* in one and was negative in the other patient. In these three patients (12, 16 and 19) CT scans showed no tumour regression whereas histological examination revealed extensive tumour necrosis. These findings underscore the difficulties in the clinical evaluation of response in mesothelioma.

### *Immunomonitoring*

After intrapleural administration of IL-2 a mild increase of $CD3^-56^+$, $CD3^+56^+$ and $CD3^-16^+$ lymphocytes was observed in pleural effusion as well as in peripheral blood. All other T-lymphocyte subsets remained at normal levels.

At dose levels A–D cytotoxicity assays showed a significant induction of LAK activity by pleural effusion-derived MNCs but not by peripheral blood MNCs. At dose levels E and F, LAK activity was induced in MNCs from both sites. Of note, no differences in LAK activity were seen between responders and non-responders.

### *Determination of cytokine levels*

Intrapleural as well as serum IL-2 levels were determined. Intrapleural levels were very high and correlated with the administered dose of IL-2. Intrapleural levels varied from 6–110 IU $ml^{-1}$ in group A to as high as 66 000–192 000 IU $ml^{-1}$ in group F. Serum IL-2 levels became measurable only in groups E and F and varied in the eight patients treated at those levels from $<3$ IU $ml^{-1}$ to 30 IU $ml^{-1}$. Intrapleural IL-2 levels were $<6000$ times higher than serum levels.

Intrapleural TNF-α levels varied from 50–125 pg $ml^{-1}$ in group A to 235–405 pg $ml^{-1}$ in group F. However, no clear relationship between TNF-α and IL-2 levels was observed as TNF-α levels in groups B–E varied from 292–1141 pg $ml^{-1}$.

### Discussion

In this phase I–II study on the toxicity and efficacy of intrapleural administration of IL-2 in patients with pleural mesothelioma we observed anti-tumour activity with acceptable toxicity. The response rate of 19% in 21 patients is of interest as mesothelioma is known to be refractory to treatment.

The basis of this study was the observation of a dose–response relationship in experimental tumour models (Ettinghausen *et al.*, 1986; Rosenberg *et al.*, 1987, 1989; Eggermont *et al.*, 1988). Systemic high-dose IL-2 therapy is associated with severe toxicity, which may prohibit application of doses with optimal anti-tumour effects (Herberman, 1989). Intrapleural administration of IL-2 is therefore a logical approach as it can be expected that very high local levels of IL-2 can be delivered without severe systemic adverse effects. We have demonstrated that high intrapleural levels of IL-2 are associated with mild to moderate toxicity, except in those patients treated at the highest dose level F with $36 \times 10^6$ IU $day^{-1}$. All patients in groups A–E were treated on an outpatient basis.

**Figure 1** Partial reponse of pleural mesothelioma (evaluable disease) and a mediastinal lymph node (measurable disease) metastasis in the left hemithorax. (**a**) Pretreatment CT scan. (**b**) After two cycles of IL-2.

Intrapleural administration of various biological response modifiers may have significant anti-tumour effects against intrapleural malignant disease. Uchida *et al.* (1984) reported that intrapleural instillation with the biological response modifier OK-432 significantly increased autologous tumour killing by tumour-associated large granular lymphocytes. The intrapleural instillation of natural β-interferon was reported to be effective against malignant pleural effusions by Rosso *et al.* (1988). Recently, Markowitz *et al.* (1992) reported on the efficacy of intracavity administration of α-interferon. Boutin *et al.* (1991) reported four CRs and two PRs after weekly intrapleural administration of γ-interferon in 22 patients with mesothelioma, also underlining the therapeutic potential of locoregional cytokine therapy.

Anti-tumour effects after intrapleural administration of TNF-α have been reported by Karck *et al.* (1990). Intrapleural administration $<200\,\mu g\,m^{-2}$ weekly in seven patients with malignant pleural effusion led to complete disappearance of tumour in three patients without side-effects. The same group reported that in patients with ovarian cancer and recurrent ascites intraperitoneal administration of TNF-α resulted in the disappearance of ascites in seven of nine patients.

Yasumoto *et al.* (1987) demonstrated that low intrapleural doses of IL-2 were sufficient to induce LAK activity in the pleural exudate and to reduce malignant pleural effusions. Manning *et al.* (1989) have shown that these activated lymphocytes were able to kill NK cell-resistant human mesothelioma cells.

Astoul *et al.* (1993) reported a relatively high response rate (7/15 patients) in mesothelioma patients who were treated with intrapleural IL-2 in a dose escalation study. The preponderance of responses was observed in stage I disease.

We made similar observations in our study. Significant LAK activity was displayed by MNCs collected from the pleural effusions after intrapleural administration of IL-2. No LAK activity was displayed by the peripheral MNCs, except at the highest two dose levels in groups E and F. However, LAK activity and response did not correspond with IL-2 dose level, intrapleural IL-2 level or intrapleural TNF-α level.

Immunophenotyping of intrapleural and peripheral MNCs showed some changes after intrapleural administration of IL-2. In this study, the dose of IL-2 did not correlate with response and none of the parameters investigated by immunophenotyping, cytotoxicity assays and determination of cytokine levels corresponded with the outcome of treatment.

Dosages of IL-2 in this study were tolerable up to and including level E, $18 \times 10^6$ IU day$^{-1}$. However, at level F, $36 \times 10^6$ IU day$^{-1}$, catheter-related infection was the most pronounced and dose-limiting side-effect. Others have reported neutrophil dysfunction during IL-2 administration, including decreased chemotaxis and Fc receptor expression (Murphy *et al.*, 1988; Jablons *et al.*, 1990; Klempner *et al.*, 1990). High-dose IL-2 increased the risk of bacteraemia significantly, which led to a reduction of catheter indwelling time (Richards *et al.*, 1991). These facts may explain the high incidence of catheter-related infections in our patients, particularly in group F, and after repeated administration of IL-2 at the lower doses.

It remains uncertain if and to what extent these infections have played a role in the induction of an anti-tumour response, as three of the four responders developed an empyema, but two of the five patients with an empyema had no response. In addition, IL-2 by itself induced high intrapleural levels of TNF-α in the majority of the patients treated.

We conclude that intrapleural administration of cytokines should be explored further as a new mode of treatment for pleural mesothelioma. IL-2 given intrapleurally appears to have anti-tumour activity against mesothelioma. We are aware of the difficulty in recommending a daily dose of IL-2 because of the small numbers of patients in each group and the frequent occurrence of empyema, but given the facts that (1) no correlation was found by us and by others between IL-2 dose and anti-tumour response, (2) low intrapleural IL-2 doses are sufficient to induce LAK activity by MNCs which can kill NK cell-resistant human mesothelioma cells and (3) mild toxicity was seen below $6 \times 10^6$ IU of IL-2 we suggest a daily dose of $3 \times 10^6$ IU of IL-2 for a phase II study.

### References

ALBERTS AS, FALKSON G, GOEDHALS L, VOROBIOF DA AND VAN DER MERWE, CA. (1988). Malignant pleural mesothelioma: a disease unaffected by current therapeutic maneuvers. *J. Clin. Oncol.*, **6**, 527–535.

ASTOUL P, VIALLAT JR, LAURENT JC, BRANDELY M AND BOUTIN C. (1993). Intrapleural recombinant IL-2 in passive immunotherapy for malignant pleural effusion. *Chest*, **103**, 209–213.

BOUTIN C, VIALLAT JR, VAN ZANDWIJK N, DOUILLARD JT, PAILLARD JC, GUERIN JC, MIGNOT P, MIGUERES J, VARLET F, JEHAN A, DELEPOULLE E AND BRANDELY, M. (1991). Activity of intrapleural gamma-interferon in malignant mesothelioma. *Cancer*, **67**, 2033–2037.

BUTCHART EG, ASHCROFT T, BARNSLEY WC AND HOLDEN MP. (1976). Pleuropneumectomy in the management of diffuse malignant mesothelioma of the pleura. *Thorax*, **31**, 15–24.

EGGERMONT AMM. (1989). Intracavitary immunotherapy: past, present and future treatment strategies. *Reg. Cancer Treatment*, **2**, 37–48.

EGGERMONT AMM & SUGARBAKER, PH. (1988). Efficacy of chemoimmunotherapy with cyclophosphamide, interleukin-2 and lymphokine activated killer cells in an intraperitoneal murine tumour model. *Br. J. Cancer*, **58**, 410–414.

EGGERMONT AMM, SUGARBAKER, PH, MARQUET, RL AND JEEKEL J. (1988). In vivo generation of lymphokine activated killer (LAK) activity by ABPP and interleukin-2 and their antitumour effects against immunogenic and non-immunogenic tumours in murine tumour models. *Cancer Immunol. Immunother.*, **26**, 24–30.

ETTINGHAUSEN SE AND ROSENBERG SA. (1986). Immunotherapy of murine sarcomas using lymphokine activated killer cells: optimization of the schedule and route of administration of recombinant interleukin-2. *Cancer Res.*, **46**, 2784–2792.

HERBERMAN RB. (1989). Interleukin-2 therapy of human cancer: potential benefits versus toxicity (editorial). *J. Clin. Oncol.*, **7**, 1–4.

HILLERDAL G. (1983). Malignant mesothelioma: review of 4710 published cases. *Br. J. Dis. Chest*, **77**, 321–343.

JABLONS D, BOLTON E, MERTINS S, RUBIN M, PIZZO P, ROSENBERG SA, AND LOTZE MT. (1990). IL-2 based immunotherapy alters circulating neutrophil Fc receptor expression and chemotaxis. *J. Immunol.*, **144**, 3630–3636.

KAPLAN EL AND MEIER P. (1958). Non-parametric estimations from incomplete observations. *J. Am. Stat. Assoc.*, **53**, 475–481.

KARCK U, MEERPOHL HG, PFLEIDERER A AND BRECKWOLDT M. (1990). TNF therapy of ascites and pleural effusions due to gynaecological carcinomas (abstract). *J. Cancer Res. Clin. Oncol.*, **116**, 328.

KLEMPNER MS, NORING R, MIER JW AND ATKINS MB. (1990). An acquired chemotactic defect in neutrophils from patients receiving interleukin-2 immunotherapy. *N. Engl. J. Med.*, **322**, 959.

LAW MR, GREGOR A, HODSON ME, BLOOM G AND TURNER-WARWICK M. (1984). Malignant mesothelioma of the pleura: a study of 52 treated and 64 untreated patients. *Thorax*, **39**, 255–259.

MANNING LS, BOWMAN RV, DARBY SB AND ROBINSON BWS. (1989). Lysis of human malignant mesothelioma cells by natural killer (NK) and lymphokine-activated killer (LAK) cells. *Am. Rev. Respir. Dis.*, **139**, 1369–1374.

MARKOWITZ A, THIELVOLDT D, YEOMANS A, LEVIN B, HUNTER C AND GUTTERMAN J. (1992). Beneficial effects of intracavitary interferon-α2b (IFN): a phase I study of patients with ascites or pleural effusion (abstract). *Proc. Am. Assoc. Clin. Oncol.*, **11**, 258.

MURPHY PM, LANE HC, GALLIN JI AND FAUCI AS. (1988). Marked disparity in incidence of bacterial infections in patients with acquired immunodeficiency syndrome receiving interleukin-2 or interferon-gamma. *Ann. Intern. Med.*, **108**, 36-41.

RICHARDS JM, GILEWSKI TA AND VOGELZANG NJ. (1991). Assocation of interleukin-2 therapy with staphylococcal bacteremia. *Cancer*, **67**, 1570-1575.

ROSENBERG SA. (1987). The development of new immunotherapies for the treatment of cancer using interleukin-2. *Ann. Surg.*, **208**, 121-135.

ROSENBERG SA, LOTZE MT, YANG JC, AEBERSOLD PM, LINEHAN WM, SEIPP CA AND WHITE DE. (1989). Experience with the use of high-dose interleukin-2 in the treatment of 652 cancer patients. *Ann. Surg.*, **210**, 474-485.

ROSSO R., RINALDI R, SALVATI F, DE PALMA M, CINQUEGRANA A, NICOLO G, ARDIZZONI A, FUSCO U, CAPACCIO A AND CENTOFANI R. (1988). Intrapleural natural beta-interferon in the treatment of malignant pleural effusions. *Oncology*, **45**, 253-256.

UCHIDA M, MISCHKE M AND HOSHINO T. (1984). Intrapleural administration of OK-432 in cancer patients: augmentation of autologous tumours killing activity of tumours-associated large granular lymphocytes. *Cancer Immunol. Immunother.*, **18**, 5-12.

WORLD HEALTH ORGANIZATION. (1979). Handbook for Reporting Results of Cancer Treatment. WHO: Geneva.

YASUMOTO K, MIYAZAKI K, NAGASHIMA A, ISHIDA T, KUDA T, YANO T, SUGIMACHI K AND NOMOTO K. (1987). Induction of lymphokine-activated killer cells by intrapleural instillations of recombinant interleukin-2 in patients with malignant pleurisy due to lung cancer. *Cancer Res.*, **47**, 2184-2187.